# Supplementary material for: Exserohilum turcicum Alters Phyllosphere Microbiome Diversity and Functions—Implications for Plant Health Management
Source: Microorganisms. 2025 Feb 27;13(3):524. doi: 10.3390/microorganisms13030524 (PMC11944305; doi:10.3390/microorganisms13030524)
Supplement: Supplementary file 1 [file microorganisms-13-00524-s001.zip › microorganisms-3473166-supplementary.pdf]

Supplementary table

Table S1 Diversity evaluation of the metagenome analysis from the phyllosphere of the adaxial and adaxial leaf surfaces of wild-type (WT) and transgenic (OE) maize.

Sample Sites	CKWT-adaxial	EWT-adaxial	CKWT-abaxial	EWT-abaxial
	Uploading Information			
bp Count	10824340373	10495960371	9887670864	11345995241
Sequence Count	71684373.33	69509671.33	65481264	75139041.33
Mean Sequence Length	151	151	151	151
Mean GC Percent (%)	52.4	55.2	52.9	55.5
	Post QC Information			
bp Count	10443297718	66951600.67	9519769320	72362067.33
Sequence Count	69482259.33	10068256648	63314571.3	10886455790
Mean Sequence Length	150	150	150	150
Mean GC Percent (%)	52.2	54.8	52.6	55.1

CKWT-adaxial, adaxial surfaces of leaves from wild-type maize; EWT-adaxial, adaxial surfaces of leaves from wild-type maize that inculated *Exserohilum turcicum*. CKWT-abaxial, abaxial surfaces of leaves from wild-type maize; EWT-abaxial, abaxial surfaces of leaves from wild-type maize that inculated *Exserohilum turcicum*.

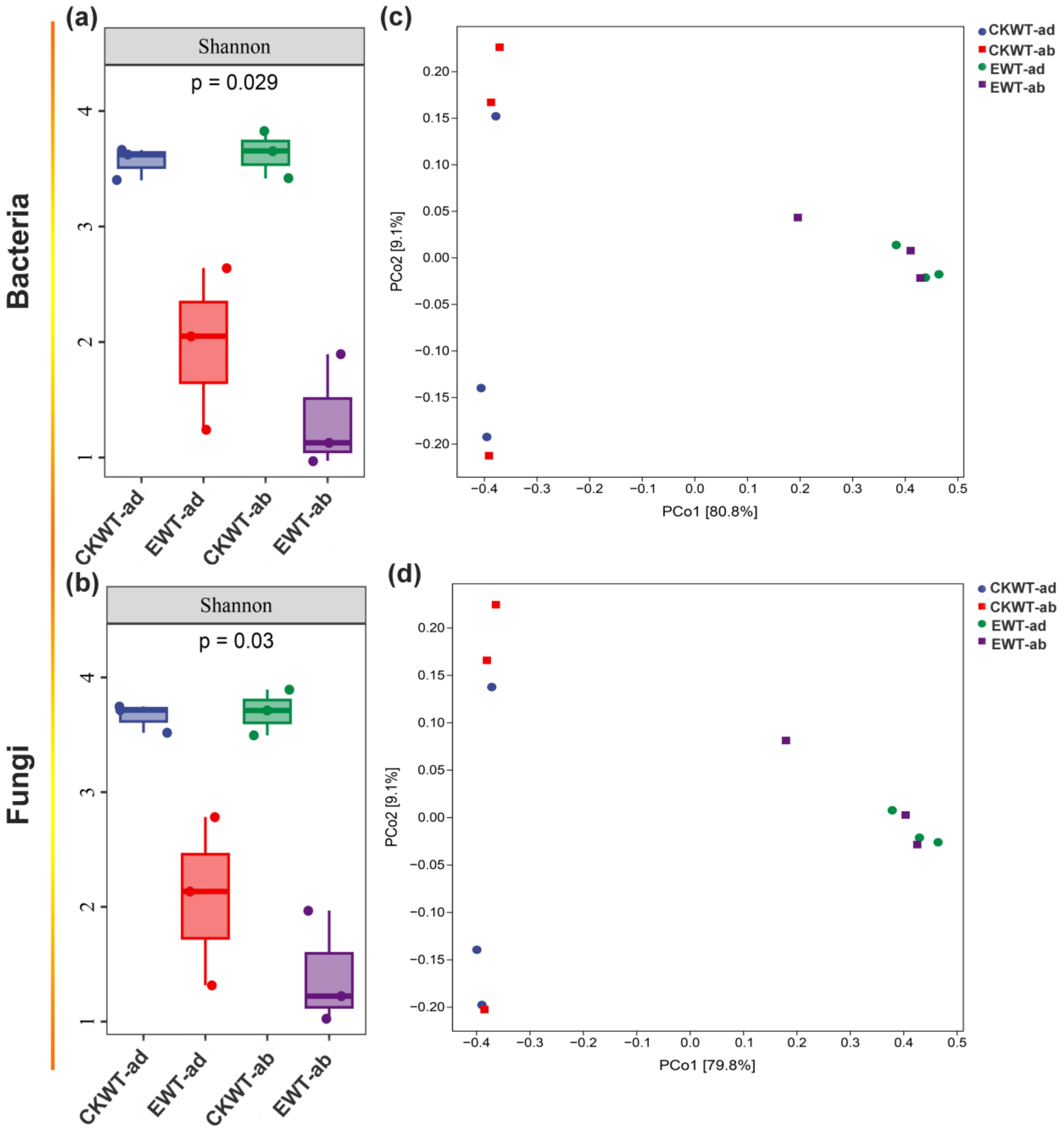

Figure S1. Bacterial and fungal communities in the maize phyllosphere. Shannon index of (a) bacterial and (b) fungal communities on the adaxial and abaxial surfaces of maize leaves (one-way analysis of variance (ANOVA), n = 3, P < 0.05). Principal coordinates analysis (PcoA) ordinations based on the Bray-Curtis similarity revealed differences in the structure of (c) bacterial and (d) fungal communities on the adaxial and abaxial surfaces of CKWT and EWT leaves (permutational ANOVA (PERMANOVA), n = 3, P < 0.05). CKWT-ad represents adaxial surfaces of wild-type maize leaves, CKWT-ab represents abaxial surfaces of wild-type maize leaves; EWT-ad represents adaxial surfaces of wild-type maize leaves that inculated *Exserohilum turcicum*, EWT-ab represents abaxial surfaces of wild-type maize leaves that inculated *Exserohilum turcicum*.

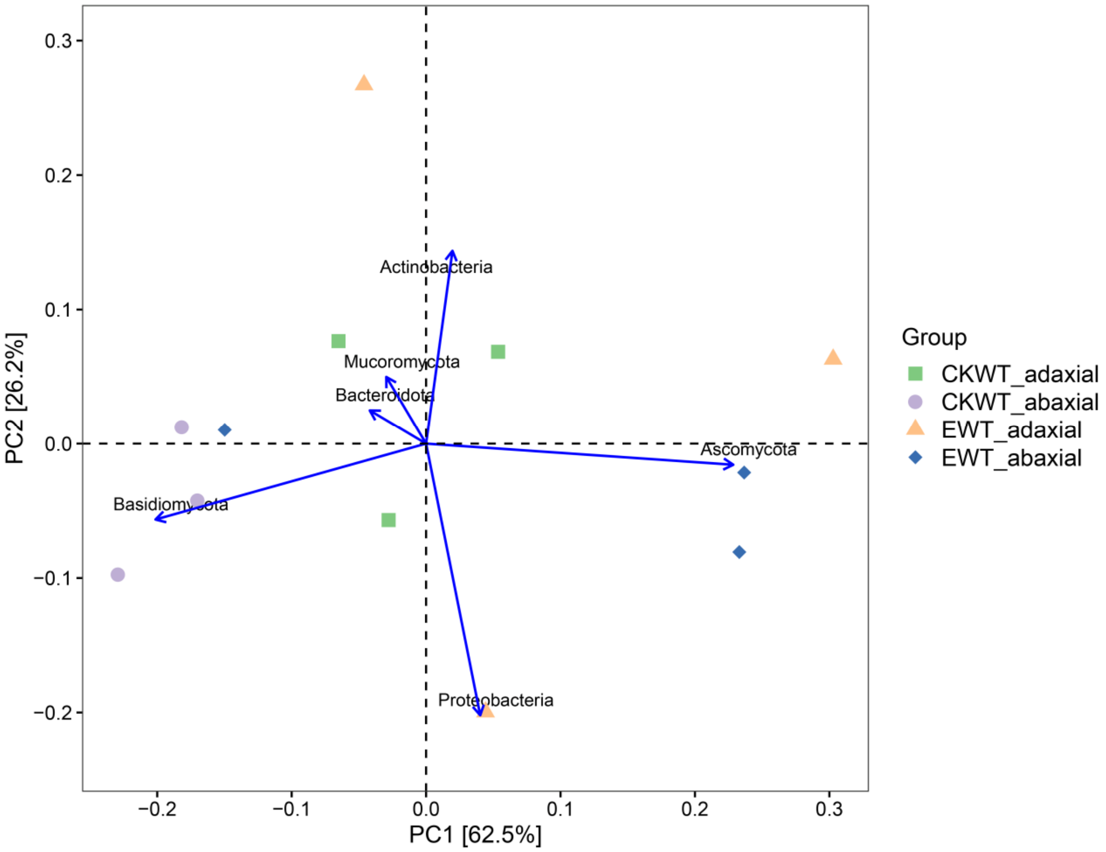

Figure S2. Principal component analysis (PCA) analysis of phyllosphere microbial community phyla. CKWT-ad represents adaxial surfaces of wild-type maize leaves, CKWT-ab represents abaxial surfaces of wild-type maize leaves; EWT-ad represents adaxial surfaces of wild-type maize leaves that inculated *Exserohilum turcicum*, EWT-ab represents abaxial surfaces of wild-type maize leaves that inculated *Exserohilum turcicum.*
